# Brief Report: Intimate Partner Violence and Antiretroviral Therapy Initiation Among Female Sex Workers Newly Diagnosed With HIV in Zambia: A Prospective Study

**DOI:** 10.1097/QAI.0000000000001841

**Published:** 2018-08-16

**Authors:** Catherine E. Oldenburg, Katrina F. Ortblad, Michael M. Chanda, Magdalene Mwale, Steven Chongo, Catherine Kanchele, Nyambe Kamungoma, Andrew Fullem, Till Bärnighausen

**Affiliations:** * Francis I. Proctor Foundation, Department of Ophthalmology, University of California, San Francisco, CA;; † Department of Global Health and Population, Harvard T.H. Chan School of Public Health, Boston, MA;; ‡ John Snow, Inc, Lusaka, Zambia;; § John Snow, Inc, Boston, MA;; ‖ Heidelberg Institute of Global Health (HIGH), Medical Faculty and University Hospital, University of Heidelberg, Heidelberg, Germany;; ¶ Africa Health Research Institute (AHRI), Somkhele, South Africa; and; # Department of Global Health and Population, Harvard T.H. Chan School of Public Health, Boston, MA.

**Keywords:** female sex workers, intimate partner violence, antiretroviral therapy, Zambia

## Abstract

**Background:**

Intimate partner violence (IPV) is common among female sex workers (FSW) globally. Here, we prospectively assessed the relationship between IPV and engagement in HIV care in a cohort of FSW who had been newly diagnosed with HIV.

**Methods:**

Data arose from the Zambian Peer Educators for HIV Self-Testing study, a randomized controlled trial of HIV self-test distribution among FSW in 3 transit towns in Zambia. Participants were self-reported to be HIV-uninfected or unaware of their status at baseline. IPV in the previous 12 months was assessed at baseline. At 4 months, participants were asked the results of their last HIV test and those who reported testing positive were asked whether they had engaged in HIV-related care and started antiretroviral therapy (ART).

**Results:**

Of 964 participants, 234 (24.3%) reported that they were living with HIV at 4 months. Of these 234 participants, 142 (60.7%) reported a history of IPV (past 12 months) at baseline and at 4 months, 152 (65.0%) reported that they were in HIV-related care, and 132 (56.7%) reported that they had initiated ART. Participants who reported IPV had significantly reduced odds of engagement in care (adjusted odds ratio = 0.48, 95% confidence interval: 0.26 to 0.91) and ART initiation (adjusted odds ratio = 0.40, 95% confidence interval: 0.22 to 0.72).

**Conclusions:**

FSW living with HIV in Zambia reported very high rates of IPV. Structural and individual interventions for violence prevention are urgently needed to better protect this population. Given the strong negative relationship between IPV and engagement in HIV-related care, such interventions could also substantially improve HIV-related health outcomes.

## Abstract 2

Supplemental Digital Content is Available in the Text.

## INTRODUCTION

Antiretroviral therapy (ART) initiation reduces HIV transmission to uninfected partners in serodiscordant couples and improves clinical outcomes for individuals living with HIV.^1–4^ Among women, intimate partner violence (IPV), which includes physical and sexual violence by a current or former intimate partner, may undermine engagement in HIV care and treatment.^5^ There may be several potential pathways through which experiences of partner violence lead to reduced engagement in HIV-related care and treatment. First, IPV may lead to depression and post-traumatic stress disorder, which can lead to reduced self-protective behaviors, increased risk of HIV acquisition, and decreased engagement in care or adherence to treatment.^6–10^ Second, women in violent partnerships may have reduced agency and reduced ability to seek care.^11,12^ Third, women living with HIV may fear disclosure of their serostatus to their violent partner, and seeking care and treatment for HIV could place them at additional risk.^11^

Female sex workers (FSW) have increased risk of HIV acquisition as a result of complex individual-, interpersonal-, and structural-level dynamics.^13,14^ FSW are at an elevated risk of IPV, both from commercial and noncommercial romantic partners, which may contribute to their increased risk of HIV acquisition as well as HIV-related outcomes.^15,16^ A substantial proportion of FSW living with HIV are not currently engaged in care or virally suppressed.^17–19^ The existing evidence of the effect of IPV on HIV care outcomes among FSW is mixed, with 1 study reporting lower risk of detectable viral load among FSW experiencing IPV,^15^ and another reporting an association between violence from sexual partners and worse outcomes.^20^ Here, we aimed to add to this literature by evaluating this association in a cohort of FSW in Zambia who were self-reported to be HIV-uninfected or unaware of their status at baseline who were participating in a randomized controlled trial of HIV self-testing.^21^

## METHODS

The Zambian Peer Educators for HIV Self-Testing study was a 3-arm cluster randomized trial designed to evaluate the effectiveness of 2 health systems delivery approaches for HIV self-test distribution among FSW in Zambia.^21,22^ FSW in Kapiri Mposhi, Chirundu, and Livingstone, Zambia, were recruited by peer educators. Eligible participants reported exchanging sex for money or goods at least once in the past month, were at least 18 years of age at the time of enrollment, were self-reported to not know their HIV status or to be HIV-uninfected, and had not recently (<3 months) tested for HIV. Participants were randomized in groups defined by the peer educator by whom they were recruited to 1 of 3 intervention arms: (1) direct delivery of the HIV self-test to the participant from the peer educator, (2) receipt of a coupon from the peer educator that could be exchanged for an HIV self-test at a participating clinic or pharmacy, or (3) referral to standard HIV testing services available in the town of recruitment. Participants completed 4 peer educator visits over the 4-month study and 3 quantitative assessments: at enrollment (baseline) and months 1 and 4 after their first study visit with the peer educator. All interviews were conducted via face-to-face interview with the same research assistant at all time points via computer-assisted personal interview in a private location that was convenient for the participant. Here, we report outcomes from the 4-month assessment to maximize the amount of time for individuals to link to care.

### Exposure Assessment

At baseline, participants were asked about past 12-month physical and sexual IPV. Physical IPV was assessed by asking whether any sexual partner had hit, slapped, punched, pushed, shoved, or performed anything else to physically hurt the participant in the previous 12 months. Sexual IPV was assessed by asking whether any sexual partner had ever physically forced them to have sex when they did not want to. If a participant responded yes, they were asked their relationship to the perpetrator (client, primary, or casual partner; participants were allowed to report multiple partner categories) separately for physical and sexual abuse.

### Outcome Assessment

At the 4-month assessment, participants were asked whether they had ever had an HIV test, when their most recent HIV test was, and the result of that HIV test. Those who reported that their most recent HIV test was positive were asked when they first learned they had HIV. All participants reporting a positive test were asked whether they were currently receiving care for their HIV and whether they were receiving ART. At 4 months, all participants were offered an HIV rapid test. At the time of the study, Zambia was following 2016 HIV treatment guidelines, which included immediate treatment eligibility for all individuals testing positive regardless of CD4 count or viral load.

### Covariates

Baseline covariates included age at enrollment, literacy, average monthly income, whether the respondent owned a mobile phone, educational attainment (dichotomized as none/primary versus some secondary or more), age at sexual debut, whether the respondent had a primary partner (defined as a longer-term sexual partner, such as a husband or a boyfriend), number of clients on an average night, and age at initiation of sex work.

### Ethics

The study was reviewed and approved by the institutional review boards at the Harvard T.H. Chan School of Public Health in Boston, MA, USA, and ERES Converge in Lusaka, Zambia. All participants provided written informed consent.

### Statistical Methods

Baseline characteristics of the study population were described with medians and interquartile ranges (IQRs) for continuous variables and proportions for categorical variables. To assess the association between baseline IPV and (1) linkage to care and (2) initiation of ART at 4 months, we used logistic regression models adjusted for potential confounding variables, including age, literacy, income, mobile phone ownership, educational status, age at sexual debut, primary partnership status, number of clients, age at initiation of sex work, time since HIV diagnosis, study site, and randomization arm.^16^ Covariates were selected based on being theoretical confounders of the relationship between IPV and HIV-care–related outcomes.^16,23–27^ We first modeled any IPV (physical and/or sexual). Second, we modeled physical IPV and sexual IPV separately. Some participants had experienced both physical and sexual IPV. In models of physical IPV, participants were coded as having experienced physical IPV if they reported any physical IPV, regardless of their experience of sexual IPV. A similar strategy was used for sexual IPV. Models of initiation of ART were among all participants (eg, those who reported not linking to care were included in the denominator). We additionally assessed the association between IPV and ever HIV testing and HIV status as assessed by the rapid test in the entire study population. All analyses were conducted in Stata 14.1 (StataCorp, College Station, TX).

## RESULTS

From September to October 2016, 965 women were enrolled in the trial, of whom 898 (93.1%) were retained at 4 months. Of these, 234 (24.3%) women reported that they tested HIV-positive over the course of the study and responded to questions related to linkage to care and ART initiation. Among these 234 women that comprised the analytic sample, 142 (60.7%) reported experiencing IPV in the past 12 months at baseline. Table 1 lists the baseline characteristics among women reporting and not reporting IPV at baseline. Among women reporting IPV, 86 (60.6%) reported both physical and sexual IPV, 30 (21.1%) reported only sexual IPV, and 25 (17.6%) reported only physical IPV. Clients were more often reported as perpetrators of IPV than nonclients.

**TABLE 1.**
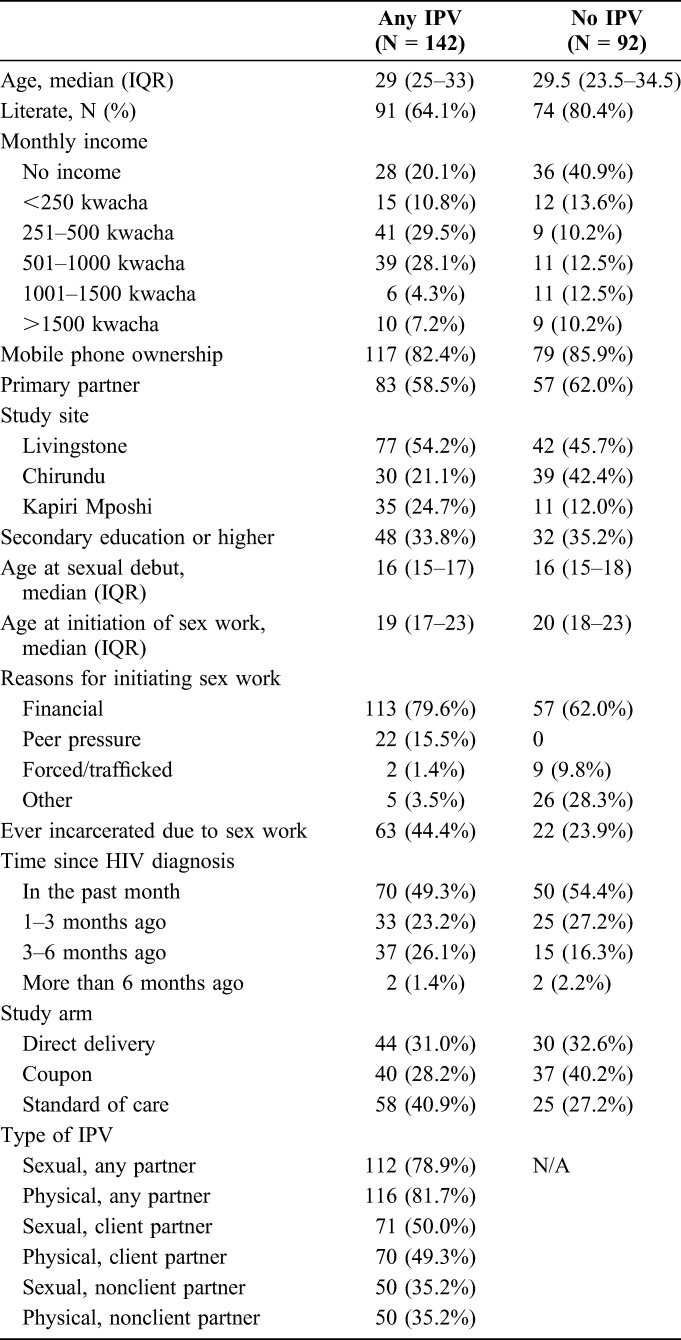
Baseline Characteristics of the Study Sample (N = 234)

**FIGURE 1.**
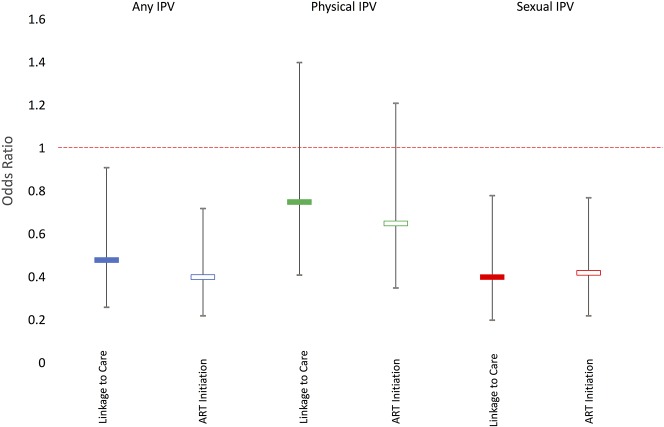
Odds ratios for engagement in HIV-related care (filled box) and ART initiation (empty box), by history of any IPV (dark gray boxes) and by physical (light gray boxes) and sexual (black boxes) IPV history.

The majority (65.0%) of participants reported that they had sought care for HIV, and approximately half (56.8%) had initiated ART. Among women experiencing any form of IPV, 59.2% reported linking to care compared with 73.9% of those who did not report IPV. Among women experiencing IPV, 48.6% reported initiating ART compared with 69.2% of those who did not report IPV. Figure 1 shows odds ratios for linkage to care and ART initiation outcomes among women reporting IPV and by type of IPV (sexual and physical). Overall, participants who reported any IPV had reduced odds of linkage to care [adjusted odds ratio (aOR) 0.48, 95% confidence interval (CI): 0.26 to 0.91, *P* = 0.03] and ART initiation (aOR 0.40, 95% CI: 0.22 to 0.72, *P* = 0.002; Table 1, Supplemental Digital Content http://links.lww.com/QAI/B208). There was no association between reporting physical IPV and linkage to care outcomes, but women who reported sexual IPV had reduced odds of both linkage to care (aOR 0.40, 95% CI: 0.20 to 0.78, *P* = 0.007) and ART initiation (aOR 0.42, 95% CI: 0.22 to 0.77, *P* = 0.005). IPV by nonclient partners was associated with greater reduced odds of linkage to care and ART initiation than by client partners (Table 2, Supplemental Digital Content http://links.lww.com/QAI/B208). In the primary trial outcome, nearly all participants reported testing for HIV.^21^ In the entire study population, there was no association between IPV at baseline and having tested for HIV at the 4-month study assessment (aOR 1.03, 95% CI: 0.54 to 1.98, *P* = 0.92) or HIV infection at 4 months (aOR 1.12, 95% CI: 0.66 to 1.89, *P* = 0.69).

## DISCUSSION

In this study, we demonstrate that past experiences of IPV negatively affect engagement in HIV-related care among FSW in Zambia. Specifically, sexual IPV was associated with approximately 60% relative decrease in the odds of linking to care or initiating ART. Sexual violence among FSW has been shown to be associated with HIV prevalence and other health outcomes, including mental health outcomes.^28^ Sexual partner violence has been shown to be associated with decreased adherence to ART among FSW who had initiated ART.^20^ Although the previous study reported no association in taking ART and IPV, this study expands on previous work by using prospective data among women who were not known to be living with HIV at baseline and evaluating the short-term uptake of HIV care. These results suggest that in the short term after HIV diagnosis, IPV experiences may be associated with decreased care-seeking behavior.

The burden of past-year IPV was very high in this population, with nearly two-thirds of participants reporting IPV in the previous 12 months at the baseline assessment. This estimate was higher than has been reported among women in the general population in Zambia, in which 18% and 10% of women in the most recent DHS reported physical and sexual IPV, respectively. HIV prevention interventions among FSW that include a violence mitigation component have been shown to reduce the burden of violence against FSW in India.^29^ For FSW living with HIV, interventions that include components related to violence victimization may help reduce the burden of violence against FSW and address barriers in accessing care related to HIV. However, additional structural interventions, including violence against women and gender norms, decriminalization, stigma-reducing interventions,^30^ and interventions with perpetrators of violence that address the overall burden of violence against FSW may be required to ultimately reduce the burden of violence and improve HIV-related outcomes in this population.

The results of this study must be considered in the context of several limitations. We did not measure emotional IPV. Emotional abuse may also influence engagement in HIV care, but we are unable to comment on it with this study. All measures included in this study were self-reported and thus may be subjected to social desirability or other biases. Participants may have underreported or overreported IPV history or linkage to care and ART initiation. Both being engaged in HIV-related care and ART initiation were high given the duration of the study and may have been overreported, although estimates were not dissimilar from a previous population-based study of FSW in Zimbabwe.^26^ We did not measure IPV at follow-up visits, and thus, we do not know how IPV may have changed over time. We did not measure adherence or viral suppression in this study, although given the short follow-up time of the study, measures of initiation of care may be more relevant.

In this prospective study, experiences of IPV were negatively associated with HIV-related care among FSW in Zambia. In the short term, IPV may slow or block engagement in care, which could result in longer-term adverse HIV-related outcomes. Development of structural- and individual-level interventions to reduce the high burden of IPV in this population is urgently needed. Such interventions may also lead to improved HIV-related outcomes and reduce HIV transmission.

## Supplementary Material

SUPPLEMENTARY MATERIAL
