# Supplementary material for: Brief Report: Intimate Partner Violence and Antiretroviral Therapy Initiation Among Female Sex Workers Newly Diagnosed With HIV in Zambia: A Prospective Study
Source: J Acquir Immune Defic Syndr. 2018 Aug 16;79(4):435–9. doi: 10.1097/QAI.0000000000001841 (PMC6203637; doi:10.1097/QAI.0000000000001841)
Supplement: SUPPLEMENTARY MATERIAL [file qai-79-435-s001.docx]

**Supplemental Table 1.** Full adjusted model results for effect of intimate partner violence on linkage to care and ART initiation at four months among FSWs living with HIV

|  | **Any IPV** | | **Physical IPV** | | **Sexual IPV** | |
| --- | --- | --- | --- | --- | --- | --- |
|  | *Link to Care* | *ART Initiation* | *Link to Care* | *ART Initiation* | *Link to Care* | *ART Initiation* |
| IPV variable | 0.48 (0.26 to 0.91) | 0.40 (0.22 to 0.72) | 0.75 (0.41 to 1.40) | 0.65 (0.35 to 1.21) | 0.40 (0.20 to 0.78) | 0.42 (0.22 to 0.77) |
| Age | 1.05 (0.10 to 1.10) | 1.01 (0.96 to 1.07) | 1.04 (0.99 to 1.10) | 1.01 (0.96 to 1.07) | 1.05 (0.99 to 1.10) | 1.01 (0.96 to 1.07) |
| Literacy | 0.94 (0.40 to 2.20) | 0.88 (0.40 to 1.89) | 1.02 (0.44 to 2.34) | 0.97 (0.46 to 2.06) | 0.89 (0.38 to 2.07) | 0.86 (0.41 to 1.82) |
| Income | 0.91 (0.71 to 1.16) | 0.83 (0.66 to 1.05) | 0.91 (0.71 to 1.16) | 0.83 (0.65 to 1.04) | 0.93 (0.73 to 1.19) | 0.85 (0.67 to 1.07) |
| Primary partner | 1.28 (0.60 to 2.72) | 1.35 (0.68 to 2.67) | 1.29 (0.61 to 2.71) | 1.31 (0.67 to 2.55) | 1.28 (0.60 to 2.71) | 1.35 (0.69 to 2.64) |
| Site  Livingstone  Kapiri Mposhi  Chirundu | 1.00  2.86 (1.21 to 6.76)  0.87 (0.30 to 2.51) | 1.00  1.81 (0.83 to 3.98)  0.93 (0.34 to 2.53) | 1.00  3.18 (1.34 to 7.51)  0.88 (0.30 to 2.58) | 1.00  2.04 (0.94 to 4.44)  0.92 (0.33 to 2.55) | 1.00  3.15 (1.36 to 7.29)  1.00 (0.34 to 2.88) | 1.00  2.07 (0.95 to 4.50)  1.04 (0.38 to 2.86) |
| Study arm  Standard  Direct  Fixed | 1.00  0.47 (0.19 to 1.18)  0.63 (0.26 to 1.54) | 1.00  0.51 (0.21 to 1.24)  0.72 (0.31 to 1.67) | 1.00  0.51 (0.21 to 1.25)  0.70 (0.29 to 1.67) | 1.00  0.53 (0.22 to 1.29)  0.80 (0.35 to 1.83) | 1.00  0.46 (0.19 to 1.12)  0.59 (0.24 to 1.43) | 1.00  0.52 (0.22 to 1.24)  0.72 (0.32 to 1.64) |
| Education | 0.47 (0.21 to 1.05) | 0.46 (0.22 to 0.96) | 0.48 (0.23 to 1.04) | 0.49 (0.24 to 0.99) | 0.44 (0.19 to 1.00) | 0.44 (0.21 to 0.93) |
| Mobile phone ownership | 0.88 (0.38 to 2.02) | 0.98 (0.43 to 2.23) | 0.91 (0.39 to 2.11) | 1.06 (0.47 to 2.40) | 0.85 (0.39 to 1.87) | 0.94 (0.43 to 2.06) |
| Age at sexual debut | 1.01 (0.88 to 1.15) | 1.05 (0.93 to 1.18) | 1.01 (0.89 to 1.14) | 1.04 (0.93 to 1.18) | 1.02 (0.89 to 1.16) | 1.06 (0.94 to 1.20) |
| Time since HIV diagnosis | 1.39 (0.89 to 2.17) | 1.36 (0.91 to 2.03) | 1.38 (0.88 to 2.15) | 1.34 (0.89 to 2.02) | 1.40 (0.88 to 2.21) | 1.36 (0.90 to 2.05) |
| Age at initiation of sex work | 1.00 (0.99 to 1.00) | 1.00 (0.99 to 1.00) | 1.00 (0.99 to 1.00) | 1.00 (0.99 to 1.00) | 1.00 (0.99 to 1.00) | 1.00 (0.99 to 1.00) |
| Number of clients on an average night | 1.07 (0.95 to 1.20) | 1.04 (0.95 to 1.14) | 1.06 (0.85 to 1.19) | 1.04 (0.85 to 1.13) | 1.08 (0.96 to 1.21) | 1.04 (0.95 to 1.15) |

**Supplemental Table 2.** Multivariable logistic regression results for effect of intimate partner violence on linkage to care and ART initiation at four months among FSWs living with HIV, by partner type

|  | Client Partner | | Non-Client Partner | |
| --- | --- | --- | --- | --- |
|  | aOR (95% CI) | P-value | aOR (95% CI) | P-value |
| *Linkage to care* |  |  |  |  |
| Physical intimate partner violence | 0.59 (0.28 to 1.27) | 0.18 | 0.49 (0.22 to 1.09) | 0.08 |
| Sexual intimate partner violence | 0.54 (0.28 to 1.02) | 0.06 | 0.36 (0.16 to 0.78) | 0.01 |
| *ART initiation* |  |  |  |  |
| Physical intimate partner violence | 0.57 (0.27 to 1.21) | 0.14 | 0.70 (0.32 to 1.50) | 0.35 |
| Sexual intimate partner violence | 0.54 (0.27 to 1.09) | 0.09 | 0.47 (0.22 to 1.00) | 0.05 |
